# Replication/Assembly Defective Avian Flavivirus With Internal Deletions in the Capsid Can Be Used as an Approach for Living Attenuated Vaccine

**DOI:** 10.3389/fimmu.2021.694959

**Published:** 2021-08-04

**Authors:** Yu He, Xiaoli Wang, Jiaqi Guo, Li Mao, Senzhao Zhang, Tao Hu, Mingshu Wang, Renyong Jia, Dekang Zhu, Mafeng Liu, Xinxin Zhao, Qiao Yang, Ying Wu, Shaqiu Zhang, Juan Huang, Sai Mao, Xumin Ou, Qun Gao, Di Sun, Yunya Liu, Ling Zhang, Yanling Yu, Anchun Cheng, Shun Chen

**Affiliations:** ^1^Institute of Preventive Veterinary Medicine, Sichuan Agricultural University, Chengdu, China; ^2^Research Center of Avian Disease, College of Veterinary Medicine, Sichuan Agricultural University, Chengdu, China; ^3^Key Laboratory of Animal Disease and Human Health of Sichuan Province, Sichuan Agricultural University, Chengdu, China

**Keywords:** live attenuated vaccine, assembly deficient, avian Tembusu virus, capsid protein deletion, protective immunity, immune response, replication deficient

## Abstract

Avian Tembusu virus (TMUV) is a novel flavivirus causing severe egg drop and fatal encephalitis in avian in Asia. In the present study, we screened the structural and functional requirements of TMUV capsid protein (CP) for viral morphogenesis using reverse genetics methods in combination with replicon packaging assays. TMUV-CP showed dramatic functional and structural flexibility, and even though 44 residues were removed from the N-terminus, it was still capable of packaging replicon RNA; in addition, 33 residues were deleted from the C-terminus (containing nearly the entire α4-helix), and infectious particles were still produced, although α4-α4’ is supposedly vital for CP dimerization and nucleocapsid formation. We further analyzed two mutants (ΔC20-43 and ΔC64-96 viruses) with relatively large deletions that still replicated well in BHK-21 cells. Our data indicate that internal deletions within CP impaired viral replication or assembly, resulting in attenuated virus proliferation in cells and attenuated virulence in duck embryos, and these deletion mutations are quite stable in cell culture. An *in vivo* assay indicated that both ΔC20-43 virus and ΔC64-96 virus were highly attenuated in ducklings but still immunogenic. Single-dose immunization with ΔC20-43 virus or ΔC64-96 virus could protect ducklings from a lethal challenge with good antigen clearance. Together, our data shed light on replication/assembly defective TMUV with internal deletions in CP and provide an effective approach to attenuate viral virulence in live vaccines without changing the antigen composition.

## Introduction

Tembusu virus (TMUV) is a newly emerged virus that was first isolated from mosquitos in Malaysia in 1955 and belongs to the *Flavivirus* genus, *Flaviviridae* family. Flaviviruses pose a significant threat to global public health, including dengue virus (DENV), Zika virus (ZIKV), yellow fever virus (YFV), Japanese encephalitis virus (JEV), West Nile virus (WNV), and tickborne encephalitis virus (TBEV). Most of these are arboviruses transmitted by mosquitoes or ticks, causing various diseases in animals and humans. Since the first outbreak of severe duck egg-drop syndrome caused by duck TMUV in mainland China in 2010 (1, 2), this virus has quickly spread to other avian species, causing enormous economic losses. Currently, it is one of the main pathogens in the poultry industry of mainland China.

Flaviviruses are a group of single-strand, positive-sense RNA viruses with a viral shell constituted by 180 copies of glycosylated E and M proteins. In the virion core are nucleocapsid complexes (NCs), containing 1 copy of viral genomic RNA (vRNA) and multiple copies of capsid protein (CP). The vRNA (~11 kb) contains a single open reading frame encoding 3 structural proteins (C, prM, E) and 7 nonstructural (NS) proteins (NS1, NS2A/2B, NS3, NS4A/4B and NS5). Structural proteins form viral particles, and nonstructural proteins participate in viral replication/assembly processes and immune escape (3, 4).

Flavivirus CP is a very small protein, only ~120 aa in length, but performs multiple roles in the viral life cycle. There is a hydrophobic anchor sequence at the C-terminus of CP, serving as a signal sequence for translocation of the prM protein. After cleavage by the NS2B-3 proteasome, mature CP (mC) is released from the anchor. The mC is approximately 100 amino acids in length. The resolved structures of different flavivirus CPs indicate that mC contains 4 distinct α helices, and similar structures and properties are shared by all flavivirus CPs (5–8). Homodimerized mC is the basic unit for NC formation, and CP dimerization is absolutely required for assembly (9, 10). This dimerized structure is mainly stabilized by pairings of α2-α2’ and α4-α4’ (9). The hydrophobic α2-α2’ interface is associated with lipid droplets, and this interaction is essential for viral particle formation (11). Positively charged α4 is proposed as a vRNA-binding site; in addition, α4-α4’ is critical for preserving the overall stability of CP dimer formation, and an unstable α4-α4’ helix pair would result in the formation of the dimer being prevented (10). Furthermore, the flexible N-terminus and α1- helix may regulate the conformation exchange of CP for various physiological processes (12). Dysfunction of CP results in the production of subviral particles (SVPs) at the expense of infectious virions (13–15).

Previous studies indicated that introducing deletions into the hydrophobic sequence (covering α2 and α3) of flaviviruses is a practical method to attenuate flaviviruses (13, 16, 17). In the present study, based on the predicted secondary structure of TMUV-CP, we carefully screened the functional requirements of TMUV-CP for viral morphogenesis by introducing a series of internal deletions covering the entire CP using a reverse genetics method in combination with a replicon packaging assay. Our data indicate that the structural integrity of TMUV-CP does not precisely correlate with its function; it can tolerate large internal deletions, truncations of up to 44 residues from the N-terminus, and nearly the entire α4-helix can be removed and viral morphogenesis still occurs. Two mutants (ΔC20-43 virus and ΔC64-96 virus) with relatively large internal deletions were highly attenuated *in vitro* and *in vivo*, but single-dose immunization with ΔC20-43 virus and ΔC64-96 virus induced protective immunity in animals. Our data provide evidence that the ΔC, internal deletion viruses, provide a mechanism to attenuate virulence *in vivo* for live attenuated TMUV vaccine generation.

## Methods and Materials

### Cells and Viruses

Baby hamster kidney cells (BHK-21) were cultured in Dulbecco’s modified Eagle’s medium (DMEM) (Gibco, Shanghai, China) supplemented with 10% fetal bovine serum (FBS) (Gibco, New York, USA) and incubated at 37°C with 5% CO2.

TMUV strain CQW1 (KM233707.1) was rescued form an infectious clone reported by our lab (18), which is an early train isolated in 2013. A more recent epidemic strain CHN-YC (MN966680.1) is isolated in 2019 (a gift from Professor Rui Luo, Huazhong Agricultural University). All virus stocks were prepared on BHK-21 cells.

### Plasmid Construction and Virus Rescue

**Figure 2:**
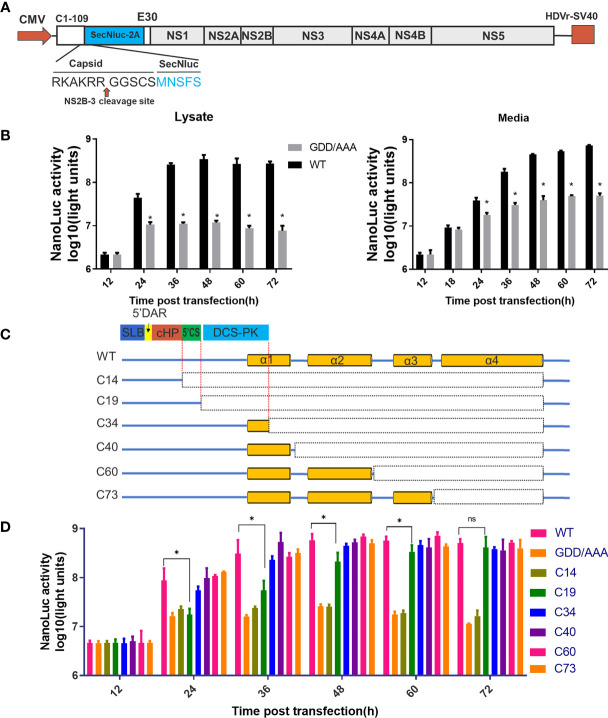
Predicted cis-elements in capsid gene are necessary for vRNA replication. **(A)** The schematic diagram for a DNA based replicon expressing secretory Nluc (mC-Replicon-SecNLuc). 1-109 aa of CP and last 30 residues of E protein were retained. **(B)** Luciferase kinetics of mC-Replicon-SecNLuc and negative-control NS5-GDD/AAA replicon on BHK-21 cells. **(C)** The schematic diagram for replicons with different truncated TMUV-CPs. **(D)** Luciferase kinetics of replicons with different truncated CPs on BHK-21 cells. Three independent experiments are presented as means and SEM, with significance defined by P value < 0.05 (*).

A DNA-based full-length infectious clone (pACNR-CQW1-Intron) for TMUV CQW1 strain (18) was used to construct all the ΔC mutants. Fragments containing the deletion mutations were engineered by overlapping-PCR, and assembly into pACNR CQW1-Intron by using *Mlu*I and *Xho*I. **Figure 2A** depict the construction strategy for a DNA-based Replicon expressing a secretory NanoLuc (SecNluc) gene. Based on a replicon plasmid (mC-Replicon-Nluc) reported previously by our lab (19), Nluc gene was substituted by SecNluc, and generated mC-Replicon-SecNLuc plasmids. All plasmids used for transfection were purified using an Endo-free Plasmid Mini Kit II (Omega Bio-tek, Georgia, USA).

To rescue the recombinant viruses, BHK-21 cells were seeded in 12-well plates, after cultured 16h, when cells were 70–90% confluence, 1.6μg plasmids were transfected into cells using TransIntro EL Transfection Reagent (TransGen Biotech, Beijing, China) per the manufacturer’s instructions.

### Replicon Assay and Nluc Activity Assay

For replicon assay, 0.2μg of wild-type (WT) or ΔC mutant replicon plasmids were transfected into BHK-21 cells seeded in 96-well plates. Every 12h, the cells were washed once with PBS and lysed using Glo lysis buffer (Promega, WI, USA) at room temperature for 5 min. The cell lysates were stored at −20°C or directly subject to Nluc activity assay.

A Nano-Glo Luciferase Assay System (Promega) was used to detect NLuc activity according to the manufacturer’s instructions. 20 μL of the sample and 100 μL of Nano-Glo Luciferase Assay Reagent was added into a white 96-well tissue culture plate. After the solution was mixed for optimal consistency, luminescence was detected using a GloMax Navigator System (Promega).

### Replicon Based Packaging Assay

To construct an efficient packaging system for evaluating the effect of capsid mutants on viral assembly, a replicon (ΔC-Replicon-Nluc) was designed as shown in **Figure 3C**. The 39-104 aa of capsid gene replaced with a gene cassette containing Nluc and FMDV 2A, the first 38 aa and last 16 aa of CP were retained. A plasmid pCDNA3.1-CprM or pCDNA3.1-mC encoding the polyprotein C-prM or mature CP were used to supply CP *in trans*.

For packaging assay, total 0.4μg of ΔC-Replicon-Nluc and WT pCDNA3.1-CprM/pCDNA3.1-mC (or various C mutant constructs) plasmids (at ratio of 1:3) were co-transfected into BHK-21 cells (each well) seeded in a 48-well plate. At 6 h post transfection, the medium was replaced with fresh maintenance medium. At 3 days post infection (dpi), the supernatant containing single-round infectious particles (SRIPs) was collected to infect with fresh BHK-21 cells. After 1.5 h incubation, the inocula were removed and washed twice with PBS, then maintenance medium was added. At 24h or 30h post infection, cells were lysed for Nluc activity assay as described above.

### Indirect Immunofluorescence (IFA)

IFA was performed as reported by our lab with slightly modified (19). Briefly, cells were washed with phosphate-buffered saline (PBS) twice, fixed with 4% paraformaldehyde for 30min, and then permeabilized for 1 h at 4°C with 0.3% Triton in PBS. After 1 h incubation at 37°C in a blocking buffer containing 5% bull serum albumin (BSA) in PBS, cells were treated with anti-TMUV mouse polyclonal antibody (Self-prepared) for 2 h and then incubated with goat anti-mouse IgG conjugated with FITC (Thermo Fisher Scientific, Shanghai, China) for 1 h. Finally, cells were stained with DAPI (Coolaber, Beijing, China) in PBS for 10 min. Each step was followed by washing the cell thrice with ice-cold PBST (1‰ Tween-20 in PBS) for 5 min in an orbital shaker. Fluorescence images were acquired under a fluorescence microscope (Nikon, Tokyo, Japan).

### Viral Titration, Growth Curve and Plaque Assay

Viral titer is determined by median tissue culture infective dose (TCID_50_) method in BHK-21 cells as reported by our lab (19).

To measure viral growth curves, BHK-21 cells were seeded in 24-well plate, after 16h cell culture (90% confluence), cells were washed with PBS twice, and incubated with WT or recombinant virus at 250 TCID_50_. After 1.5 h attachment at 37°C, the inocula were removed. Afterward, the cells were washed twice with PBS, and supplied with DMEM containing 2% FBS and 1% penicillin/streptomycin. the supernatant was collected every 12 h, and subjected to viral titration.

Plaque assay was performed as described previously with slight alteration (20). Viral samples were 10-fold serially diluted in DMEM; 300μl samples of each dilution were added to a 12-well plate seeded with BHK-21 cells at approximately 95% confluence. After 1.5h attachment at 37°C, 1 ml of 1% methyl cellulose overlay containing 2% FBS and 1% penicillin/streptomycin was added to each well, and the plate was incubated for 5 days. Then, overlay was removed, the plate was washed twice with PBS, fixed with 4% formaldehyde at room temperature for 20 min, then stained with 1% crystal violet for 1 min. Finally, the cells were washed carefully, and visible plaques were observed.

### Animal Experiments

To determine the immune response after immunization, 25-day-old ducks (purchased from Waterfowl Breeding Center of Sichuan Agriculture University) were intramuscular injected with 200μL (10^5^ TCID_50_) WT virus or F10 of ΔC viruses (11 ducks each group), mock group were incubated with equal volume of DMEM. At 3 and 5 dpi, three ducks in each group were euthanized, serum samples and tissue samples including heart, liver, spleen, lung, kidney, brain, and thymus were collected for viral tissue loads detection. The rest of ducks were raised for monitoring survival rate for 14 days. At 14 days post immunization, ducks were bled for T-lymphocyte proliferation assay, IFN-γ and IL-4 in serum were also analyzed by ELISA. Meanwhile, serum samples were collected weekly for 7 weeks to monitor the neutralization antibodies.

Virulence and preventive effect of ΔC20-43 virus and ΔC64-96 virus as vaccine candidates were evaluated using 5-day-old Specific pathogen Free (SPF) ducklings. SPF duck embryos were purchased from the Harbin Veterinary Research Institute (China), incubated in 37°C incubators. Ducklings were transfer to isolators with negative pressure as soon as they hatched. 5-day-old SPF ducklings were intramuscular injected with 200 μL (at a dose of 10^5^ TCID_50_) WT, F10 of ΔC20-43 virus or F10 of ΔC64-96 virus. Mock-infected ducklings were given DMEM. Ducklings were monitored for weight change every two days; survival and signs of disease were checked daily. At 3 days post immunization, ducklings were bled to determine viremia in BHK-21 cells. At 14 days post immunization, ducklings were challenged with (a currently epidemic TMUV strain) CHN-YC (2×10^6^ TCID_50_). On day 3 post-challenge, ducklings were bled and viremia was determined. After challenge, weight change, survival and signs of disease were monitored. Survival percentage were record until 14 days post challenge.

### T-Lymphocyte Proliferation Assay, Detection of IFN-γ and IL-4 by ELISA

The peripheral blood lymphocyte proliferation assay (21) was performed using a modified CCK8 method. Briefly, T-lymphocytes were isolated using a Peripheral Blood Lymphocyte Separation Kit (Solarbio, Beijing, China) according to manufacturer’s instruction. After cell counting, 80 μL diluted cells in RPMI 1640 medium (Gibco) were seeded into a 96-well plate. To specifically stimulate the proliferation of T-lymphocytes, 20 ul CHN-YC virus (10^6.25^ TCID_50_/100 ul) or purified recombinant truncated E protein (20 mg/mL) were added; for mock group, equal volume of PBS was added. After 36 h cell cultured in 37 °C, cell proliferation was detected using a Cell Counting Kit-8 (MCE, Shanghai, China) per manufacturer’s instruction.

The expression of IFN-γ and IL-4 measured by ELISA. Th1-type cytokine IFN-γ and Th2-type cytokine IL-4 in serum at 14 dpi were measured using commercial duck IFN-γ and IL-4 sandwich ELISA kits (mlbio, shanghai, China) following the manufacturer’s instruction.

### Plaque Reduction Neutralization Test (PRNT)

Neutralizing antibody in serum were determined by PRNT as described previously (22). Serum was inactivated at 56°C for 30 min, and 5-fold continuous diluted to 5^-7^with DMEM. Diluted samples were mixed with an equal volume of WT CQW1 virus (120 TCID_50_); for virus control groups, equal volume of DMEM were mixed with virus. Then mixtures were incubated at 37°C for 1 h, and then distributed into 12-well plates seeded BHK-21 cells. Subsequent procedures of plaque assay as described in above. The effective dilution of sera to 50% end point titers (NT_50_) was calculated using the Kärber method.

### RNA Extraction and RT-qPCR

The total RNA was isolated using RNAiso Plus regent (Takara, Dalian, China) per manufacturer’s instruction. To measure vRNA level in tissues or the transcriptional expression of cytokines in spleens, reverse transcription quantitative PCR (RT-qPCR) assays were performed using 2×Taq SYBRGreen qPCR Premix (Innovagene, Changsha, China) in a CFX Connect Real-Time PCR Detect System (Bio-rad) following the manufacturer’s protocols. Primers used in present study are presented in **Table S1**.

### Quantification and Statistical Analysis

Data of the Nluc activities, RT-qPCR, ELISA and viral titers are presented as means ± Standard Error (SEM). Student’s t-test was used to assess statistical significance, with significance defined by P value <0.05 (*) in GraphPad Prism 8.0 software. Statistical significance of survival was analyzed using survival curve, Log-rank (Mantel-Cox) test in GraphPad Prism 8.0 software, with significance defined by P value <0.05 (*).

### Ethics Statement

All animal experimental procedures were approved by the Institutional Animal Care and Use Committee of Sichuan Agriculture University in Sichuan, China (Protocol Permit Number: SYXK(川)2019-187).

## Results

### Predicted RNA Secondary Structure and Protein Secondary Structure of Capsid

**Figure 1:**
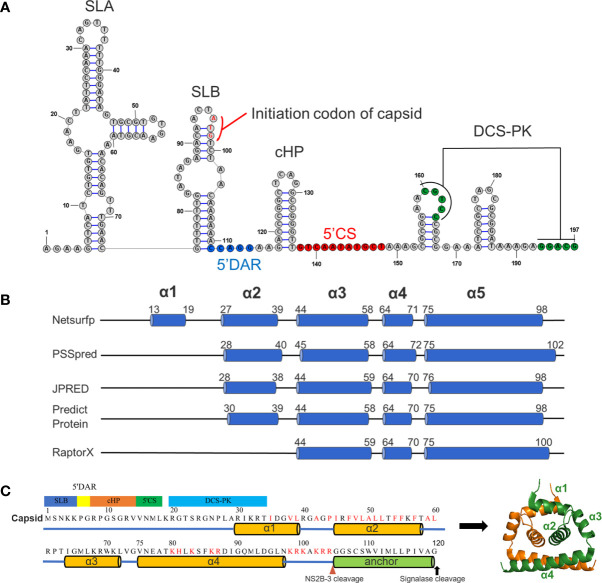
Predicted RNA secondary structure and protein secondary structure for TMUV capsid. **(A)** Predicted RNA secondary structure for TMUV 1-200 nt using RNAstructure 6.0.1. The capsid gene (95-197nt) involves in SLB, 5’DAR, cHP, 5’CS and DCS-PK. **(B)** The results of predicted secondary for TMUV-CP, using different bioinformatics algorithms. **(C)** A model for TMUV-CP. TMUV-CP contains four α helices and a transmembrane helix (termed as anchor), and two mature CPs form a dimer (right).

Previous studies have shown that CP plays multiple roles in the flaviviral life cycle. Its major function is the formation of the NC core, which participates in the viral assembly process, but it is also involved in vRNA replication *via* conserved RNA secondary structures in the N-terminus of the capsid gene (12). Therefore, first, the RNA secondary structure of the CQW1 capsid gene was predicted *via* homologous modelling using RNAstructure 6.0.1. In this model (**Figure 1A**), the first 100 nucleotides of the capsid gene are involved in the formation of SLB, 5’DAR and 5’CS, cHP and DCS-PK, and all of these cis-elements are conserved in the *Flavivirus* genus and are significant for vRNA replication (12).

We next analyzed the secondary structure of mature CP (1-104 aa) using several bioinformatics algorithms (Netsurfp, PSSpred, JPRED, Predict Protein and RaptorX). At least three α-helices were consistently predicted with minor variations in the length and position of the α-helix by all programs (**Figure 1B**). Combined with previously resolved CP structures derived from DENV (7), WNV (8), ZIKV (6, 23) and JEV (5), we finally proposed a structural model for TMUV-CP (**Figure 1C**). In this model, similar to other flaviviruses, mC of TMUV folds into 4 distinct α-helices (α1-α4), but its N-terminus forms an unstructured region; the anchor sequence in the C-terminus of CP also forms a transmembrane α-helix; and two TMUV-CPs form a homodimer. These data will be used throughout the present study.

### Capsid Genes Containing Cis-Elements Are Necessary for vRNA Replication

The flaviviral replicon is a convenient and efficient tool to measure viral translation and replication (24). In the present study, a DNA-based replicon (mC-Replicon-SecNLuc) was used to verify the functional requirements of the conserved RNA elements in the capsid gene. A schematic diagram for replicon construction is shown in **Figure 2A**. The characteristics of this replicon were verified in BHK-21 cells. As shown in **Figure 2B**, after transfection, the Nluc activities of the replication-deficient NS5-GDD/AAA replicon reached a plateau at 24 h post infection in the cell lysate, representing the initial viral translation levels. By comparison, the Nluc activities of the WT replicon rapidly increased over time, reached a peak at 48 h post infection, and generated approximately 30-fold Nluc activities in excess of the NS5-GDD/AAA replicon. These Nluc activity differences between these two groups represent viral replication. High Nluc activities were also detected in the cell medium, and both groups increased over time at all indicated timepoints; presumably, Nluc was extremely stable after secretion in the supernatant compared with intracellular levels.

Based on the predicted RNA secondary structure of the capsid gene, different replicon constructs with a series of truncated CPs were generated (**Figure 2C**). Then, we assessed the replication kinetics of these replicons in BHK-21 cells (**Figure 2D**). After transfection, most constructs (C34, C40, C60 and C73) showed indistinguishable replication kinetics when compared with WT. The C19 replicon showed impaired replication at early timepoints compared with the WT replicon but reached a similar peak at later periods. C14 showed a similar curve to the NS5-GDD/AAA replicon. These results indicate that only the first 19 aa of CP is sufficient to support vRNA replication, while the first 34 aa of TMUV-CP ensure the full replication capability of the replicon, suggesting that the DCS-PK element accelerates viral replication and that 5’CS is key for vRNA replication. In addition, the results also indicate that there are no other cis-elements in the rest of the region of the TMUV capsid gene. These properties should be taken into consideration when constructing replicon tools, inserting tags into the 5’ UTR and capsid gene or generating internal deletions within CP to generate ΔC infectious clones in our subsequent studies.

### TMUV-CP Shows Remarkable Functional Flexibility in Viral Morphogenesis

To better characterize the functional importance of the secondary structures of TMUV-CP on viral morphogenesis, a DNA-based full-length infectious cDNA clone for the CQW1 strain (18) was used to generate various ΔC mutants. After transfection into BHK-21 cells (to generate F0 viruses), the proliferation of these mutants was verified by IFA.

**Figure 3:**
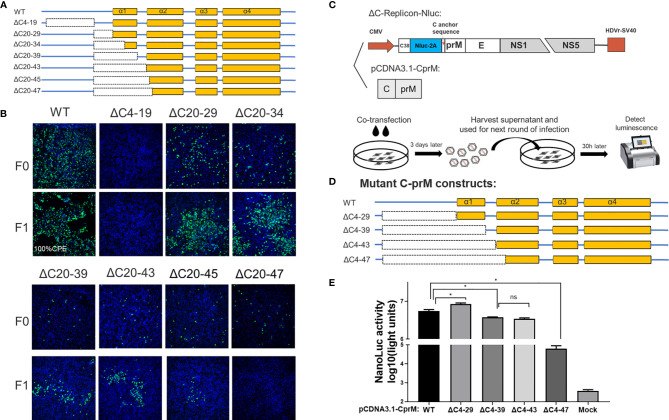
The N-terminus of TMUV-CP showed a remarkable functional flexibility. **(A)** The schematic diagram for mutant infectious clones with different deletions at the N-terminus to α1 of CP. **(B)** The viability of ΔC mutants with truncation at the N-terminus to α1, determined by IFA on BHK-21 cells. F0 viruses were generated by directly transfection with infectious clone plasmids on BHK-21 cells. **(C)** The schematic diagram of the DNA based packaging system. This system contains two components, the ΔC-replicon (with CP deleted, C38 means the first 38 residues of CP) expressing Nluc and the eukaryotic expression plasmid pCDNA3.1-CprM supplementing CP *in trans*, respectively. And the experimental designs shown in the bottom. **(D)** Mutant C-prM constructs used for packaging assay performed in the **(E)**. **(E)** The effect of the N-terminus of CP on viral assembly, determined by packaging assay. Three independent experiments are presented as means and SEM, with significance defined by P value < 0.05 (*).

A set of seven deletions was introduced into the flexible N-terminus and α1 of CP, as shown in **Figure 3A**. At 5 days post transfection, a strong fluorescence signal was detected in WT samples; by contrast, a moderate signal was observed in most of the other mutants, and the signal was reduced with the deletion size; an exception was the ΔC4-19 mutant, which did not produce any fluorescence, indicating that its viral replication was abolished (**Figure 3B**). To further validate the viability of these mutants, the supernatant of these mutants was harvested at 5 days post transfection and used to infect fresh BHK-21 cells to generate F1 viruses. At 3 dpi, the presence of infectious virions was detected by IFA (**Figure 3B**). The ΔC20-47 mutant with a large deletion is incapable of producing infectious particles under these experimental conditions. Other mutants with smaller deletions were viable but exhibited varying degrees of impairment, even though α1 was completely deleted.

Although the replication-deficient ΔC4-19 mutant is not infectious, we cannot conclude that the unstructured N-terminus is involved in viral morphogenesis because this region is essential for RNA replication. Therefore, to verify this possibility, we used a packaging system (as shown in **Figure 3C**) to evaluate the effect of the N-terminus on the viral assembly/release process. In this system, the C-prM polyprotein was supplemented *in trans* to package ΔC-replicon-Nluc. Interestingly, compared with the WT-CprM construct, a minor enhanced packaging efficiency (1.5-fold) was observed when 4-29 residues were removed (**Figures 3D, E**), and a similar result was reported in a previous study for YFV (25). Slightly decreased packaging efficiencies were observed for ΔC4-39 and ΔC4-43; moreover, ΔC4-47 showed a significantly decreased efficiency (90-fold) compared with WT, generating a basic level of Nluc activity (**Figures 3D, E**). Altogether, these results indicate that the N-terminus and α1 of CP are not indispensable for viral morphogenesis; 44 residues were removed from the N-terminus, while CP retained the ability to package replicon RNA.

**Figure 4:**
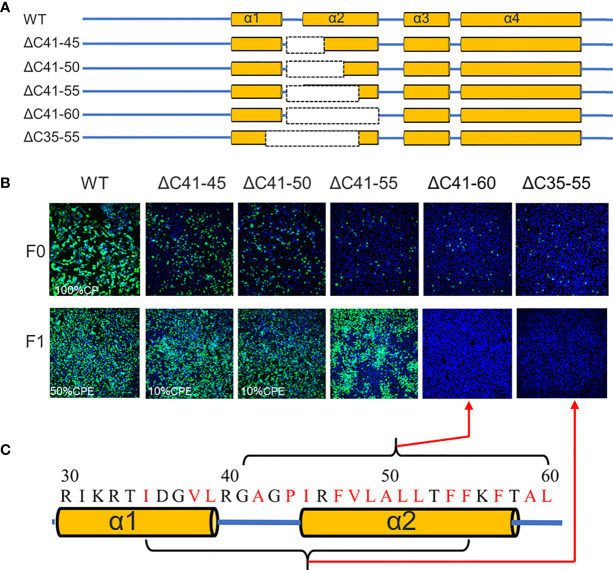
The α2 of TMUV-CP is less tolerated to internal deletion. **(A)** It depicts the mutant infectious clones with different deletions at the α2-helix of CP, and their viabilities are verified on BHK21 cells by IFA using anti-TMUV polyclonal antibody as the primary antibody **(B)**. **(C)** The hydrophobic residues located at α2-helix.

Previous studies have demonstrated that flavivirus internal hydrophobic sequences are essential for correct viral assembly (14, 25, 26). In the present study, α2 (44-58 aa) was fully located within the TMUV internal hydrophobic sequence (approximately 35-60 aa). Four deletions (ΔC41-45, ΔC41-50, ΔC41-55 and ΔC41-60) were introduced into α2 of TMUV CP (**Figure 4A**). All mutants replicated in BHK-21 cells, but their viability was slightly impaired, and only ΔC41-60 was completely abolished (**Figure 4B**). To validate that the number of hydrophobic residues or the length of the deletion, but not certain sites within aa 56-60 of α2, caused the observed failure of viral morphogenesis, another mutant, ΔC35-55, was generated. Compared with ΔC41-55 (11 hydrophobic resides), 14 hydrophobic resides were located in both the ΔC35-55 and ΔC41-60 regions (**Figure 4C**). As expected, ΔC35-55 also did not produce any infectious particles (**Figure 4B**). These results indicate complete removal of α2 and abolished infectious particle production, and the hydrophobic amino acids within α2 may be responsible for this.

**Figure 5:**
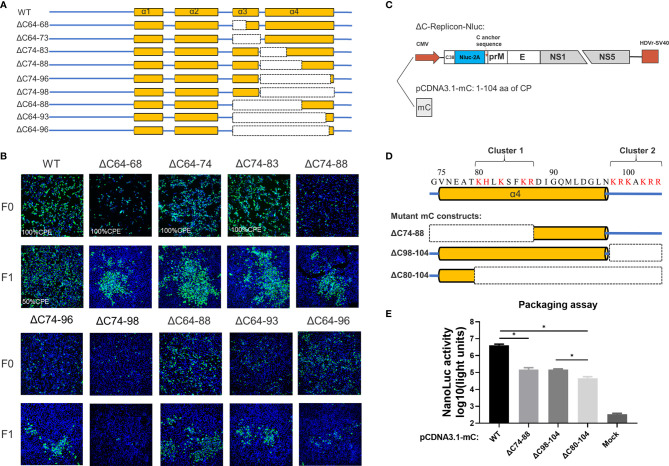
The C-terminus of TMUV-CP is responsible for vRNA binding but could tolerate large deletions. **(A)** The schematic diagram for mutant infectious clones with different deletions at the α3 to C-terminus of CP. **(B)** The viability of the ΔC mutants with deletions at the α3 to C-terminus, determined by IFA on BHK21 cells. **(C)**The packaging system used in the **(E)**, eukaryotic expression plasmid pCDNA3.1-mC encoding the mature CP was used to supplements CP *in trans*. **(D)** Two clusters of basic residues located at the α4 to C-terminus. **(E)** Effect of these two clusters on viral assembly, determined by packaging assay. Three independent experiments are presented as means and SEM, with significance defined by P value < 0.05 (*).

**Figure 6:**
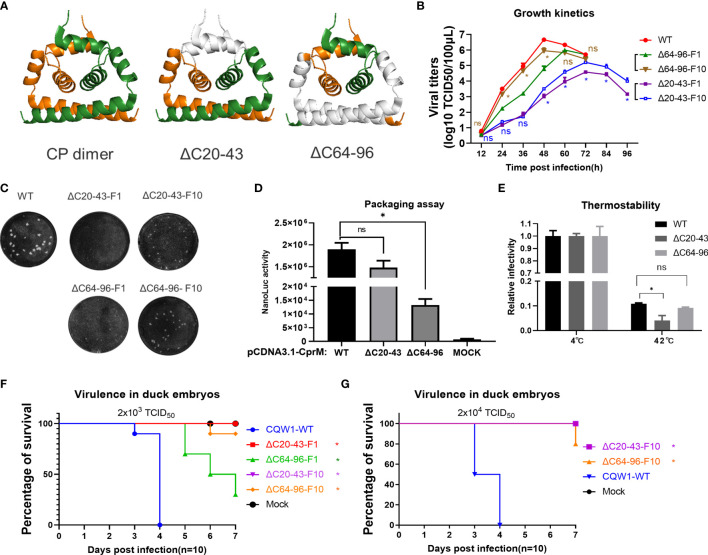
Characteristics of the ΔC20-43 virus and ΔC64-96 virus *in vitro*. **(A)** The structural models of wild-type TMUV-CP dimer, ΔC20-43 dimer and ΔC64-96 dimer. The deleted regions were indicated in white color. **(B)** Growth kinetics and **(C)** plaque morphology of the ΔC20-43 and ΔC64-96 viruses on BHK-21 cells. **(D)** Effect of the ΔC20-43 and ΔC64-96 deletions on virus assembly, which were determined by packaging assay on BHK-21 cells. **(E)** Thermostability of the ΔC20-43 and ΔC64-96 viruses. Three independent experiments are presented as means and SEM, with significance defined by P value <0.05 (*). Virulence of the ΔC20-43 and ΔC64-96 viruses in duck embryos at a dose of **(F)** 2,000 TCID_50_ or **(G)** 20,000 TCID_50_. Statistical significance of survival was analyzed using survival curve, Log-rank (Mantel-Cox) test, with significance defined by P value < 0.05 (*).

Next, we introduced a set of deletions into the α3 and α4 regions of CP (**Figure 5A**). Similar to WNV (13), α3 is dispensable for viral morphogenesis, and complete removal of α3 (ΔC64-73 mutant) slightly attenuated viral production (**Figure 5B**). Although α4 has been proposed as the vRNA binding site, infectious particles were also produced by the ΔC74-96 mutant, even though nearly all α4 was deleted, but complete removal of α4 (ΔC74-98) was inviable. Given the apparent tolerance of α3 and α4 to deletion, we constructed additional truncations covering the α3 and α4 regions to determine the functional limits of CP. Surprisingly, after removal of 33 residues from the α3 and α4 regions, almost 32% (33/104) of mC (**Figures 5A** and **6A**) of the ΔC64-96 mutant still produced infectious particles, although this effect was severely attenuated (**Figure 5B**).

Analysis of the residue composition of the α4-helix in the C-terminus indicated that there were two clusters of basic residues (**Figure 5D**). The first basic cluster was located at the middle of α4 (K80, H81, K83, K86, R87), while the second basic cluster (K98, R99, K100, K102, R103, R104) was located at the end of mC outside α4. We further evaluated the effect of these two clusters on viral assembly *via* a replicon packaging assay (**Figure 5C**). WT-mC was supplemented *in trans* and generated a high level of Nluc activity at 30 h post infection; by contrast, when either cluster 1 or cluster 2 was removed, both ΔC74-88 and ΔC98-104 generated a (27-fold) lower Nluc activity than WT, and when both clusters were removed, the Nluc activity generated by ΔC80-104 was 90-fold lower than that generated by WT (**Figure 5E**). Thus, these results indicate that both clusters participate in vRNA binding, but there is still a low level of packaging efficiency upon baseline observation in ΔC80-104, suggesting that α4 at the C-terminus is not the only site for vRNA binding. Collectively, the above data demonstrated that the capsid protein tolerates large internal deletions, especially in the N- and C-termini, and it shows unexpected functional flexibility in viral morphogenesis. We summarized of outcomes of different constructs generated in this study in **Table S2**.

### Characteristics of the ΔC20-43 and ΔC64-96 Mutants *In Vitro*

A central goal of the present study is to assess whether CP could act as a target to attenuate TMUV and be used for live attenuated vaccine (LAV) development. It seems that the impaired replication of ΔC virus on BHK-21 cells corresponds to its deletion size; thus, we selected two mutants (ΔC20-43 and ΔC64-96 viruses) with relatively large deletions at different positions of CP (**Figure 6A**). To better understand the effect of ΔC20-43 and ΔC64-96 deletions on the *in vitro* characteristics of TMUV, we further analyzed ΔC20-43 and ΔC64-96 viruses on BHK-21 cells. As shown in **Figure 6B**, the proliferation of both mutants was significantly attenuated in BHK-21 cells. Compared with WT virus, both mutants reached their peak with different degrees of delay, and the peak titers of both the ΔC20-43(10^4.6^ TCID50/100 μL) and ΔC64-96 (10^5.8^ TCID50/100 μL) mutants were significantly lower than that of WT virus (10^6.7^ TCID50/100 μl, **Figure 6B**); among these two mutants, ΔC20-43 was better attenuated than ΔC64-96, perhaps because ΔC20-43 was deficient in both replication and assembly, as revealed by the replicon assay.

To test the stability of deletion mutations in CP and whether ΔC mutants might revert to a better growth phenotype in cell culture, ΔC20-43 and ΔC64-96 viruses were continuously propagated on BHK-21 cells for 10 passages. Then, ΔC20-43-F10 and ΔC64-96-F10 were subjected to whole genome sequencing. As shown in **Table 1**, even after 10 passages, the ΔC20-43 and ΔC64-96 mutations showed good viral genome stability without any other mutations in the capsid gene. This indicates large deletion within CP is very stable. In addition, other mutations were spread throughout the whole genome of both mutants. As the cell passage increased, the time required for these two mutants to cause cytopathic effects decreased and stabilized at 70 h (ΔC20-43) and 55 h (ΔC64-96); moreover, the viral titers also increased with passage. Presumably, these mutations located outside the CP may be responsible for this effect. Hence, we performed a plaque assay and determined the growth kinetics of F10 viruses of ΔC20-43 and ΔC64-96 on BHK-21 cells, to verify this possibility. As shown in **Figure 6C**, the WT virus formed significantly larger plaques than the other viruses. Plaques formed by ΔC20-43-F1 were nearly invisible, but clearly visible plaques were observed for ΔC20-43-F10; in contrast, ΔC64-96 could form small plaques at F1, and ΔC64-96-F10 formed larger plaques than F1. Corresponding to this, the F10 viruses of ΔC20-43 and ΔC64-96 replicated better than their F1 viruses, respectively, but still showed attenuation compared with WT virus (**Figure 6B**). These results demonstrate that large deletions within CP are very stable, although continuous passage resulted in a better growth phenotype in cell culture.

**Table 1:** Stability of continuous passages of ΔC20-43 and ΔC64-96 on BHK-21 cells.

ΔC20-43-F10			ΔC64-96-F10		
Location	Nucleotide Position	Amino Acid Change	Location	Nucleotide Position	Amino Acid Change
C	Deletion 153-224	deletion 20-43	C	Deletion 285-383	deletion 64-96
NS3	A6619G, A6318C	Y537C, N577H	E	A2131G, C1809T	Q392R, H285Y
NS4B	C7025T	Y44*	NS1	A2919G	K154E
NS4	G9582C	G643R	NS2A	A3893G	P126*
			NS5	G9582C	G643R
			3’UTR	C10588T	——

*Indicates silent mutation.

The main CP functions as the building block for NC assembly and plays an essential role in the assembly of infectious particles. To test the effect of ΔC20-43 and ΔC64-96 deletions on viral assembly/release in detail, a packaging assay was performed as described above. Interestingly, ΔC20-43 deletion barely affected packaging under this condition, but ΔC64-96 deletion severely impaired viral packaging (**Figure 6D**). A previous study demonstrated that α4 is a key element for the stability of CP dimers and that mutations in α4 affect the thermostability of CP dimers (10). Thus, we further evaluated the thermostability of mutant viruses. Equal doses (2,000 TCID_50_) of WT, ΔC20-43 and ΔC64-96 viruses were incubated at 42°C or 4°C for 1.5 h and then subjected to viral titration. Unexpected, only ΔC20-43 but not ΔC64-96 showed decreased thermostability (**Figure 6E**) compared with WT.

Next, we assessed the viral virulence of ΔC20-43 and ΔC64-96 in duck embryos, and F1 and F10 of both mutants were included in this assay. As shown in **Figure 6F**, duck embryos were highly sensitive to WT virus, and all embryos died within 4 days when infected with 2000 TCID_50_ (a low dose). In comparison, no embryos died within 7 days in either the ΔC20-43-F1 or ΔC20-43-F10 groups; ΔC64-96-F1 showed a fatality rate of 60%, which was significantly lower than that of WT viruses. Notably, ΔC64-96-F10 showed a fatality rate of only 10%, suggesting that adaptive mutations in the ΔC64-96-F10 genome may further attenuate its virulence in duck embryos. Therefore, we assessed the virulence of ΔC20-43-F10 and ΔC64-96-F10 at a high infective dose (**Figure 6G**). Unexpectedly, no embryos died with ΔC20-43-F10, while 80% of embryos survived the ΔC64-96-F10 challenge.

Altogether, these data indicate that internal deletions within CP impaired viral replication (ΔC20-43) or assembly (ΔC64-96), resulting in attenuated virus proliferation in BHK-21 cells and virulence in duck embryos, and these deletion mutations were quite stable in cell culture. Adaptive mutations located outside the CP in the viral genome can restore viral spread in BHK-21 cells but further attenuate the virulence of TMUV in duck embryos.

### The Immune Responses Stimulated by ΔC20-43 and ΔC64-96

**Figure 7:**
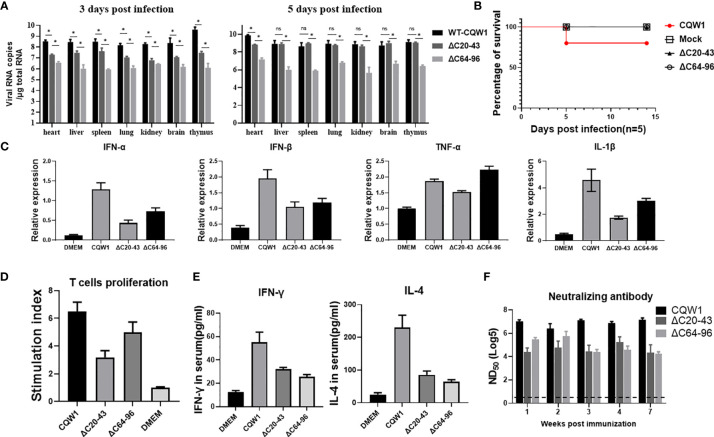
The immune responses stimulated by the ΔC20-43 and ΔC64-96 viruses. 25-day-old ducks were infected with the WT virus, ΔC20-43 and ΔC64-96 viruses at a dose of 10^5^ TCID_50_, respectively. Mock group is treated with DMEM. **(A)** The vRNA loads in heart, liver, spleen, lung, kidney, brain and thymus of ducks were detected by RT-qPCR, at 3 dpi and 5 dpi. **(B)** The percentage of survival of 25-day-old ducks post infection. **(C)** Relative mRNA expression of IFN-α, IFN-β, TNF-α and IL-1β in the spleen were detected at 5 dpi. **(D)** Duck peripheral T-lymphocytes proliferative response to TMUV infection. **(E)** At 14 dpi, the serum levels of IFN-γ and IL-4 in the ducks were determined by ELISA. **(F)** Neutralization antibodies level in the serum were determined by PRNT. Data are presented as means and SEM, with significance defined by P value < 0.05 (*), ns means no significance.

To better understand the characteristics of ΔC20-43 and ΔC64-96 viruses *in vivo*, we first evaluated the immunogenicity of the ΔC20-43 and ΔC64-96 mutants (F10 viruses) in ducks. At 3 dpi, the viral load of WT virus is significantly higher than that of both ΔC mutant viruses in all tested tissue samples, especially in the thymus, reaching 10^9.6^ copies/μg of total RNA; at 5 dpi, the number of copies further increased, reached a high titer in all tested tissues (more than 10^9^ copies/per μg of total RNA). In comparison, the viral copy number of ΔC20-43 was significantly higher than that of ΔC64-96 in all tissues at 3 dpi and further increased to a high level compared to that of the WT virus in most samples, except in the heart. However, ΔC64-96 always maintained a relatively low level of viral RNA copies in all tissues at 3 and 5 dpi (**Figure 7A**). Ducklings infected with WT-TMUV exhibited depression, anorexia and neurological signs at 4~7 days, and only one duckling died at 6 dpi (**Figure 7B**). This is not surprising because the susceptibility of ducks to TMUV infection decreases as ducks grow older (27). However, ducklings infected with both ΔC viruses did not show any obvious symptoms during the 14-day observation period.

Cellular immunity plays an important role in preventing flavivirus infection (28). To analyze the innate immune responses stimulated by ΔC viruses, we compared the abilities of WT and ΔC viruses to induce innate cytokine responses in spleen samples from infected ducks at 5 dpi (**Figure 7C**). Both ΔC viruses could induce high cytokine expression at the mRNA level; however, cytokine expression was lower than that of the WT virus, including IFN-α, IFN-β, TNF-α and IL-1β. These data indicate that innate immune response patterns induced by ΔC viruses and WT virus are similar. Next, we assessed whether cellular immunity is activated by ΔC20-43 virus and ΔC64-96 virus infection. At 14 dpi, IFN-γ (Th1-type cytokine) and IL-4 (Th2-type cytokine) in duck serum samples were analyses by ELISA. As expected, markedly higher levels of IFN-γ and IL-4 (**Figure 7E**) were detected in the WT and ΔC virus-receiving groups than in the mock (DMEM) group, although ΔC viruses induced relatively moderate cytokine levels compared to WT viruses. Then, we further evaluated the cellular immune response by determining the ability of duck peripheral T-lymphocytes to proliferate in response to TMUV infection (**Figure 7D**). Compared with the mock group, a significantly enhanced T lymphocyte proliferation response against TMUV infection was detected in samples from ducks infected with WT, ΔC20-43 and ΔC64-96. However, T lymphocytes of mock-infected ducks did not respond to TMUV infection under this experimental condition. Therefore, these results indicate that ΔC20-43 and ΔC64-96 can elicit cellular immune responses in ducks.

Given that humoral immunity is essential to eliminate flavivirus infection, we collected serum samples at the indicated timepoints to examine for the presence of neutralizing antibodies against TMUV (**Figure 7F**). Both ΔC20-43 and ΔC64-96 viruses induced detectable neutralizing antibodies at all tested timepoints and showed a downward trend from 1 week to 7 weeks post infection; ΔC64-96 infection produced higher titers of neutralizing antibodies than ΔC20-43 at early timepoints. However, compared with WT viruses, the neutralization antibodies induced by both ΔC viruses were mild. Altogether, the data in this section suggest that ΔC20-43 and ΔC64-96 viruses induce analogous immune response patterns in 25-day-old ducks and that both innate and adaptive immunity can be stimulated by their infection. Generally, ΔC64-96 infection produced a more powerful immune response than ΔC20-43 infection; however, compared with WT virus, both ΔC20-43 and ΔC64-96 viral infections induced a relatively more moderate immune response.

### Single-Dose Immunization of ΔC20-43 and ΔC64-96 Viruses Protected Ducklings From Lethal Challenge by an Epidemic TMUV

**Figure 8:**
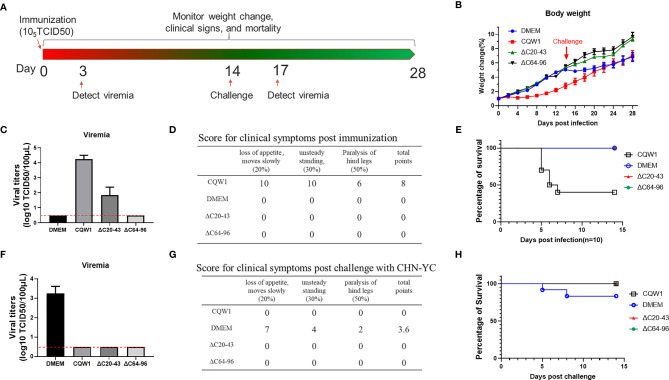
Single-dose immunization of the ΔC20-43 and ΔC64-96 viruses protected ducklings from lethal challenge by an epidemic TMUV. **(A)** Experimental design for animal experiment. 5-day-old ducklings were intramuscularly injected with the WT virus, ΔC20-43 and ΔC64-96 mutant viruses at a dose of 10^5^ TCID_50_, respectively. Mock group is treated with DMEM. At 14 days post immunization, survival ducklings were challenged with a virulent TMUV. **(B)** Weight change post immunization (days 1-14) and post challenge (days 15-28). **(C)** Viremia post immunization. **(D)** Clinical symptoms post immunization. In each item, 1 score corresponding to 1 duck appear symptom. The total points= 20% * item 1 + 30% * item 2 + 50% * item 3. **(E)** Percentage of survival post immunization. **(F)** Viremia post challenge. **(G)** Clinical symptoms post challenge. **(H)** Percentage of survival post challenge.

To better determine the virulence of ΔC20-43 and ΔC64-96 viruses *in vivo*, a more sensitive animal model was used to evaluate the virulence properties of these two ΔC viruses. **Figure 8A** outlines the experimental design. Five-day-old ducklings were first vaccinated with WT or two ΔC viruses (F10 viruses) at a dose of 10^5^ TCID_50_ *_via_* intramuscular injection. The infected ducklings were monitored for weight changes (**Figure 8B**), viraemia (**Figure 8C**), clinical symptoms (**Figure 8D**) and survival (**Figure 8E**) for two weeks. Compared with the DMEM group, no differences in weight change were observed in the ΔC20-43- and ΔC64-96 virus-infected groups, while the duckling weight of the WT virus-infected group was significantly lower than that of the DMEM group on day 4 and afterward. At 3 dpi, WT virus developed robust viraemia (reaching a titer of 10^4^ TCID_50_/100 μL), and ΔC20-43 infection also produced mild viraemia (approximately 10^2^ TCID50/100 μL). Interestingly, no viraemia was detected in the ΔC60-96-infected group during our study, which may be due to the extremely low viral titers in the ΔC60-96-infected groups below the detection baseline of our method, consistent with the low viral load in the tissues above. WT virus-infected ducklings began showing different degrees of symptoms on day 3, including depression, slow movement, loss of appetite, weight reduction, unsteady standing and even paralysis of hind legs; 60% of ducklings died from WT virus infection (3, 2 and 1 ducklings died on days 5, 6 and 7 after infection, respectively). The rest of the ducklings gradually recovered from symptoms after 7 dpi. By comparison, no obvious symptoms were observed in the ΔC20-43- and ΔC64-96 virus-infected groups or the mock (DMEM) group, and no ducklings died after immunization. Collectively, the results demonstrate that the ΔC20-43 and ΔC64-96 viruses are highly attenuated.

Next, to test whether these ΔC mutants were capable of inducing protective immunity, immunized ducklings were challenged with the virulent TMUV CHN-YC strain at 14 days post immunization. Except for the DMEM group, no viraemia was detected in the WT, ΔC20-43 and ΔC64-96 virus groups at 3 days post challenge (**Figure 8F**). Relatively milder symptoms were observed in the DMEM-immunized group post challenge (**Figure 8G**) than in the first immunization with CQW1, and only two ducklings in the mock group died from CHN-YC challenge on days 5 and 8 (**Figure 8H**). This is because 5-day-old ducklings more easily succumb to TMUV infection than 19-day-old ducklings. The weight gain of the DMEM group was significantly slower than that of the ΔC20-43 and ΔC64-96 groups post challenge (**Figure 8B**). No obvious symptoms were observed in the WT, ΔC20-43, or ΔC64-96 groups (**Figure 8G**). It is important that all ducklings survive challenge. Therefore, single-dose immunization with ΔC20-43 virus or ΔC64-96 virus can protect ducklings from lethal challenge.

## Discussion

Flavivirus CP is one of three structural proteins but is multifunctional in the viral life cycle. Although the nucleotide and amino acid sequences of flavivirus capsids are not conserved among flavivirus genera, similar structures and properties are shared by all flavivirus CPs. In the present study, we carefully analyzed the structural and functional requirements of TMUV-CP for viral replication and assembly.

In the predicted model for a secondary structure of mature TMUV-CP, there are four distinct α helices. Flaviviral CP is a structural protein and is pivotal for forming flavivirus NC, but previous studies have demonstrated that CP shows remarkable functional flexibility (13–16). However, previous studies have mainly focused on introducing deletions into the α2 helix (13, 15, 16), a region overlapping with the internal hydrophobic sequence. In the present study, we carefully defined the functional requirements of TMUV-CP using a powerful reverse genetics system in combination with a packaging assay by introducing a set of deletions covering the whole mature CP. Consistent with previous studies, TMUV-CP tolerates large deletions. α1 or α3 is dispensable for CP, and complete removal of either of them does not abort infectious particle production. However, hydrophobic α2 is less tolerant to deletions, and a mutant (ΔC40-60) with complete removal of α2 is inviable. It has been demonstrated that the α2-α2’ interface forms a hydrophobic cleft in flavivirus CP dimers. This hydrophobic cleft is supposed to interact with lipid droplets and other biological membranes and is vital for viral particle formation (11); furthermore, the pairing of α2-α2’ is also responsible for the dimerization of CP (5–7). Therefore, a deletion that is too long within this region is not practical. Meanwhile, we identified two basic residue clusters at the C-terminus of mature CP, suggesting that the last seven basic residues of mC are not only crucial for NS2B3 cleavage but also play an important role in vRNA binding. Our unpublished data also showed that a TMUV mutant with engineered acidic residues in the second cluster rapidly reverts to a basic phenotype after continuous passage in cell culture, indicating the functional requirements of this cluster. Removing either of these two clusters results in impaired packaging efficiency. Interestingly, in the packaging assay, even though these two clusters were completely removed, mutant ΔC80-104 could still package the replicon, which may be because the N-terminus of CP is also involved in vRNA binding (29). However, a mutant (ΔC74-98) with a deletion simultaneously involving the two clusters is unviable, which indicates that infectious virion assembly may be a stricter process *in situ* than in a packaging assay. A previous study indicated that α4–α4′ association is required for dimerization of CP, as mutations in α4 abrogate NC formation (10). To our surprise, the ΔC64-96 mutant containing 33-residue (104 residues in all) deletions, including all α3 residues and nearly all α4 residues, was removed and still produced infectious particles. Therefore, these data indicate that TMUV-CP tolerates large deletions in CP and suggest that the structural integrity of TMUV-CP is not very precisely correlated with its functions.

Although TMUV-CP tolerates large deletions in CP, these ΔC mutants showed various degrees of attenuation in viral spread during cell culture, corresponding to their deletion sizes. The *in vitro* growth properties of ΔC20-43 and ΔC64-96 in BHK-21 cells showed that ΔC20-43 was more attenuated than ΔC64-96 compared with WT. Although the flexible N-terminus and α1 are supposed to help CP to adopt different conformations for various physiological processes (12), the results of the packaging assay indicate that the C-prM protein with ΔC20-43 deletion has little effect on its ability to package the replicon RNA; in comparison, Δ64-96 deletion severely impairs its packaging efficiency, and it would result in secretion of non-infectious SVPs in the expense of virions (15). Besides, the results revealed by the replicon assay indicate that ΔC20-43 deletion also attenuated vRNA replication, on account of the DCS-PK element was removed in ΔC20-43 mutant (**Figure 1C**). This result suggested that the attenuated vRNA replication efficiency is more responsible for the attenuated phenotype of the ΔC20-43 mutant in cell culture. Thus, defects in vRNA replication or viral assembly are the molecular basis of ΔC20-43 and Δ64-96 showed attenuation *in vivo*.

A central aspect of this study relates to identifying CP as a new target to attenuate TMUV *in vivo* and its use for live attenuated vaccine development. Flavivirus LAVs generally offer effective and durable immunity following a single immunization, and this property is suitable for rapid vaccination on a large scale and is relatively inexpensive. In the present study, both ΔC20-43 and ΔC64-96 viruses were attenuated but still protected ducklings from a virulent TMUV challenge. ΔC viruses have similar benefits as LAVs; moreover, compared with traditional methods for LAV development, this approach has several advantages: (1) The ΔC mutant has the same antigen composition (prM/E) as the WT virus, and authentic nonstructural proteins could induce a specific immune response. (2) Compared with point mutations, deletion mutations, especially large deletions, are quite stable, and it is almost impossible to revert to a WT sequence. One possible concern is that the adaptive mutations after ΔC viruses continuously passaged on cell cultures, generated replication-enhanced phenotypes in cell culture, with potentially unknown consequences on vaccine safety. Nonetheless, in the present study, these adaptive mutations (F10 viruses) did not lead to revert to a highly virulent phenotype, as demonstrated by results of the virulence assay in duck embryos and 5-day-old ducklings; ΔC64-96-F10 virus even further attenuated in duck embryos compared with ΔC64-96-F1 virus. Similar results are observed in previously studies (13, 15). In addition, ΔC could be combined with other mutations to further increase its safety; for example, the T367K substitution in E protein (30) can be incorporated into ΔC mutants, generating a recombinant virus with a double assurance for attenuation. In the present study, the ΔC mutants showed various degrees of attenuation corresponding to their deletion sizes *in vitro*. Regarding safety as primary consideration, we chose two mutants (ΔC20-43 and ΔC64-96) with relatively large deletions as candidates for LAVs. However, the most important point is to achieve the balance between attenuation and immunological responses, too attenuated virus may be insufficient to induce an effective protective immunity. In consideration of revealed molecular basis for attenuation of ΔC virus, we probably can regulate the level of attenuation *in vivo*, by regulating the size of ΔC deletions, but this hypothesis needs more experimental evidences. Besides, although both ΔC20-43 and ΔC64-96 viruses showed good immunity for ducks in the present study, ΔC64-96 virus showed significantly lower viral loads, compared with ΔC20-43 virus, and did not cause detectable viremia in ducks. For the safety concern, ΔC64-96 virus may be better for further application, but a more extensive challenge experiments still needed to further demonstrate the efficacy and the time of duration of immune protection effect of these two ΔC viruses in the future.

To conclude, we demonstrated that TMUV-CP showed that the structural integrity of CP is not precisely relevant to its function. CP could tolerate large deletions and is a potential target for virulence attenuation. Introducing internal deletions into CP would impair viral RNA replication/assembly, resulting in attenuated phenotypes *in vitro* and *in vivo*. We demonstrated that TMUV with internal deletions in CP could be developed as single-dose LAVs demonstrating safety and immunogenicity, representing a promising platform.

## Data Availability Statement

The raw data supporting the conclusions of this article will be made available by the authors, without undue reservation.

## Ethics Statement

All animal experimental procedures were approved by the Institutional Animal Care and Use Committee of Sichuan Agriculture University in Sichuan, China (Protocol Permit Number: SYXK(川)2019-187).

## Author Contributions

YH and SC conceived and led this study. YH drafted the manuscript. SC reviewed and edited the manuscript. YH, XW, GJ, SeZ and LM performed the experiments and analyzed the data. SC, AC and WM provided the materials and funding for the study. HT, LZ, RJ, DZ, ML, XZ, QY, YW, ShZ, JH, SM, XO, QG, DS, YL and YY participated in data analysis and partial experiments. All authors contributed to the article and approved the submitted version.

## Funding

This work was funded by grants from the National Key Research and Development Program of China (2017YFD0500800), the China Agriculture Research System of MOF and MARA, and the Program Sichuan Veterinary Medicine and Drug Innovation Group of China Agricultural Research System (SCCXTD-2021-18).

## Conflict of Interest

The authors declare that the research was conducted in the absence of any commercial or financial relationships that could be construed as a potential conflict of interest.

## Publisher’s Note

All claims expressed in this article are solely those of the authors and do not necessarily represent those of their affiliated organizations, or those of the publisher, the editors and the reviewers. Any product that may be evaluated in this article, or claim that may be made by its manufacturer, is not guaranteed or endorsed by the publisher.

## Supplementary Material

Supplementary Table 1 The primers used in this study.

Supplementary Table 2 Summary of outcomes of different constructs generated in this study.
